# Comparison on the Electrochemical Corrosion Behavior of Ti6Al4V Alloys Fabricated by Laser Powder Bed Fusion and Casting

**DOI:** 10.3390/ma17133322

**Published:** 2024-07-04

**Authors:** Zhongwei Zhan, Qi Zhang, Shuaixing Wang, Xiaohui Liu, Hao Zhang, Zhihua Sun, Yulin Ge, Nan Du

**Affiliations:** 1 Aviation Key Laboratory of Science and Technology on Advanced Corrosion and Protection for Aviation Material, AECC Beijing Institute of Aeronautical Materials, Beijing 100095, China; 15101180406@126.com (Q.Z.); 15010176035@163.com (Z.S.); geyulinhoho@163.com (Y.G.); 2 School of Materials Science and Engineering, Nanchang Hangkong University, Nanchang 330063, China; xiaohuiliu612@163.com (X.L.); zhanghhao6666@126.com (H.Z.); 11004@nchu.edu.cn (N.D.)

**Keywords:** additive manufacturing, laser powder bed fusion, Ti6Al4V alloy, corrosion resistance

## Abstract

The non-equilibrium solidification process in the additive manufacturing of titanium alloy leads to special microstructures, and the resulting changes in corrosion behavior are worthy of attention. In this paper, the microstructure and electrochemical corrosion behavior of Ti6Al4V alloys prepared using laser powder bed melting (LPBF) and casting are systematically compared. The results show that the LPBF-processed Ti6Al4V alloy is composed of dominant acicular α′ martensite within columnar prior β phase, and less β disperses have also been discovered, which is significantly different from the α + β dual-phase structure of cast Ti6Al4V alloy. Compared to the as-cast Ti6Al4V alloy, LPBF-processed Ti6Al4V alloy has a thinner and unstable passive film, and exhibits slightly poorer corrosion resistance, which is mainly related to its higher porosity, a large amount of acicular α′ martensite and less β phase compared to as-cast Ti6Al4V alloy. This result proves that suitable methods should be taken to control the relative density and phase composition of LPBF-processed Ti6Al4V alloys before application.

## 1. Introduction

Titanium (Ti) and its alloys have been increasingly used in aerospace, petrochemical, biomedicine and other fields as they have an excellent combination of low density, high specific strength and outstanding corrosion resistance [1,2]. However, the traditional manufacturing methods for Ti alloy, such as casting and computer numerical control (CNC) machining, are not conducive to the processing of composite and integrated titanium alloy parts [3].

Laser additive manufacturing (LAM) technology has shown great advantages in the direct manufacturing of titanium alloy parts with complex geometry [4]. The requirements of precision forming and high performance are taken into account in LAM. Laser powder bed fusion (LPBF) is one of the powder-bed-based LAM methods using a laser to selectively melt successive layers of metal powder in an inert-gas-filled chamber [5]. LPBF can accurately control the internal structure and complex shapes and directly produce the metal parts with metallurgical bonds and high dimensional accuracy. LPBF technology has been widely applied in the manufacturing of aerospace components such as titanium alloy blisks and blades, as well as biomedical materials such as teeth and bones [6].

However, the non-equilibrium rapid solidification nucleation and growth process of titanium alloys occurs under the ultra-high temperature gradient and ultra-fast cooling rate in the LAM process. The resultant uniqueness and diversity of the microstructure (such as grain morphology and size, crystal orientation, composition uniformity, etc.) are different from that of traditional alloys. Thijs et al. found that the primary β grains of LPBF-processed Ti6Al4V alloy were significantly refined [7]. Meanwhile, the martensite α′ slabs with different crystal orientations were formed in the primary β grains due to the high cooling rate of the micro-molten pool. Dinda et al. proved that the primary β grains of additive-manufactured Ti6Al4V alloy grew epitaxially and acicular α phases were interlaced with each other in a basket-like shape [8]. A lot of dislocations and some twins were present in the α phases. Li et al. also believed that additive-manufactured Ti6Al4V alloy exhibited a finer lamellar α + β structure within the considerably coarser columnar primary β grains as compared to the traditional forged Ti6Al4V alloy consisting of equiaxed α and transformed β phases [9]. The differences in the microstructure characteristics of additive-manufactured Ti6Al4V alloys might be related to the laser equipment and process parameters used. However, as a whole, the current research on LAM of titanium alloys mainly focused on the relationship between microstructure and mechanical properties [10]. Further research is needed on the environmental adaptability and durability of additive-manufactured titanium alloys.

It is widely known that the electrochemical corrosion behavior of Ti6Al4V alloys is related to their microstructure, which depends on the processing technology. Therefore, it is urgent to study the relationship between the microstructure and electrochemical corrosion behavior of Ti6Al4V alloys prepared by different processes. Yang et al. found that among the microstructural factors influencing corrosion resistance of Ti6Al4V alloys fabricated by different AM methods, the type of constituent phase is the main one [11]. Li et al. confirmed that the chemical element composition difference between the α phase and the β phase in the Ti-6Al-4V alloy is the reason for the selective dissolution of the α phase [12]. Dai et al. found that the LPBF-processed Ti6Al4V alloy possesses poorer corrosion resistance than the Grade 5 sample, due to the considerably large amount of acicular α′ and less β–Ti phase in the microstructure of the LPBF-processed sample compared to the Grade 5 sample [13]. Note that these studies were carried out in a neutral NaCl solution [14,15,16]. Generally, titanium alloys come into contact with acidic corrosive media in certain service environments, which poses more stringent requirements for the corrosion resistance of LAM titanium alloys. However, the comparing research on the electrochemical corrosion behavior of Ti6Al4V alloys fabricated by LPBF and cast in acidic electrolytes has not been fully investigated. Specifically, there is less research on the pitting behavior of LPBF-processed Ti6Al4V alloy in acidic environments through dynamic electrochemical impedance spectroscopy (DEIS). Therefore, in order to ensure the safety, reliability and long-term service of Ti6Al4V alloys formed by LAM in the aviation environment, it is necessary to study the corrosion damage behavior and corrosion mechanism of LAM-formed titanium alloys in an acidic environment.

In the work, the microstructures, potentiodynamic curves, electrochemical impedance spectroscopy (EIS) and DEIS tests of Ti6Al4V alloys fabricated by LPBF and casting were investigated. The typical surface morphologies after the DEIS test were observed to discuss the corrosion mechanisms in the HCl solution. The results can provide data reference for predicting the corrosion and structural design of additive-manufactured Ti6Al4V alloy, and guide the selection of appropriate processes for the Ti6Al4V alloy manufacturing process.

## 2. Materials and Methods

### 2.1. Materials and LPBF Processing

**Figure 1:**
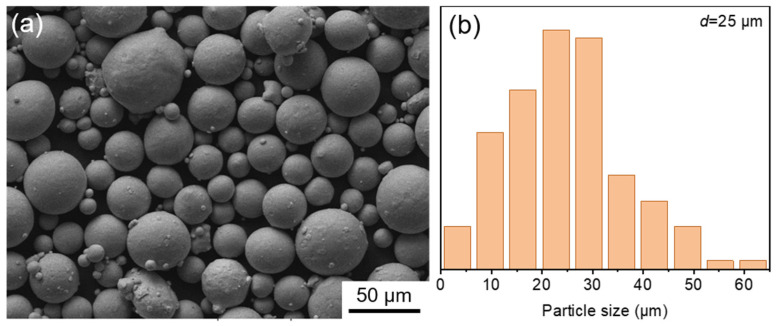
The morphology (**a**) and particle size distribution range (**b**) of Ti6Al4V alloy powders.

The morphology and particle size distribution range of Ti6Al4V alloy powders used for LPBF are shown in Figure 1. Powders are nearly spherical and accompanied by a small amount of satellite balls. The diameter of the particles is approximately 25 μm. The LPBF experiments were conducted using an EOS M280 system (EOS GmbH, Maisach, Germany). During the LPBF process, the laser powder, scan speed, hatch space and layer thickness were set as 370 W, 2100 mm/s, 80 μm and 40 μm, respectively. A scanning direction of 67 between adjacent layers was rotated, and a building platform preheating temperature of 200 °C was employed. A Ti6Al4V alloy with similar compositions was prepared by casting for comparison.

### 2.2. Microstructural Characterizations

Metallurgical defects of the as-built and as-cast alloys after polishing were observed by an optical metallographic microscope (OM, U-TV0.5XC-3, Olympus, Tokyo, Japan). The polished samples were etched with Kroll’s reagent (3 mL HF, 6 mL HNO3 and 90 mL H_2_O) for 20 s for the microstructural investigations using a scanning electron microscope (SEM, Nova Nano SEM450, Thermo Fisher Scientific, Hillsboro, OR, USA). Chemical compositions of the alloys were analyzed using energy dispersive spectroscopy (EDS, INCA 250, Oxford Instruments, Oxford, UK). The phase composition of the samples was analyzed using an X-ray diffractometer (XRD, D8-advance, Bruker, Karlsruhe, Germany) at a scan rate of 12°/min.

### 2.3. Electrochemical Measurements

The samples with an exposed area of 100 mm^2^ were prepared for the electrochemical measurements. The non-working surface of the electrode was sealed with epoxy resin. The test surface of the as-built Ti6Al4V alloy was parallel to the building direction. Electrochemical tests were conducted under a 1 mol HCl solution (pH = 1). Electrochemical tests were performed by an electrochemical workstation (Autolab PGSTAT 302N). A standard three-electrode electrochemical cell with a saturated calomel electrode (SCE) as a reference electrode, a platinum sheet as the auxiliary electrode and Ti6Al4V alloy specimens as the working electrode were carried out for the tests.

Before electrochemical testing, the samples are immersed in a 3.5% NaCl solution for approximately 30 min to stabilize the open circuit potential (OCP). The potentiodynamic polarization curves were performed from a potential range of −0.8 V (SCE) to the break of the passive film with a potential scanning rate of 1 mV/s. EIS was acquired at OCP over the frequency range of 10^−2^~10^5^ Hz using an AC signal amplitude of 10 mV. Dynamic electrochemical impedance spectroscopy (DEIS) tests were performed in the frequency range of 10^−2^~10^5^ Hz at the potential from 1.0 V (SCE) to 3.0 V (SCE) with the application of sinusoidal potentials of amplitude 10 mV. The EIS and DEIS data were fitted using ZSimpWin software, version 3.6. All electrochemical tests were conducted at room temperature (25 ± 1 °C), and to ensure reliability, each set of electrochemical tests was repeated three times. After DEIS tests, the surface corrosion morphology of the samples was observed with a three-dimensional digital video microscope (KH7700, Hirox Co., Ltd., Tokyo, Japan), and the three-dimensional morphology of corrosion pits were constructed.

## 3. Results

### 3.1. Microstructural Analysis of As-Cast and As-Built Ti6Al4V Alloys

**Figure 2:**
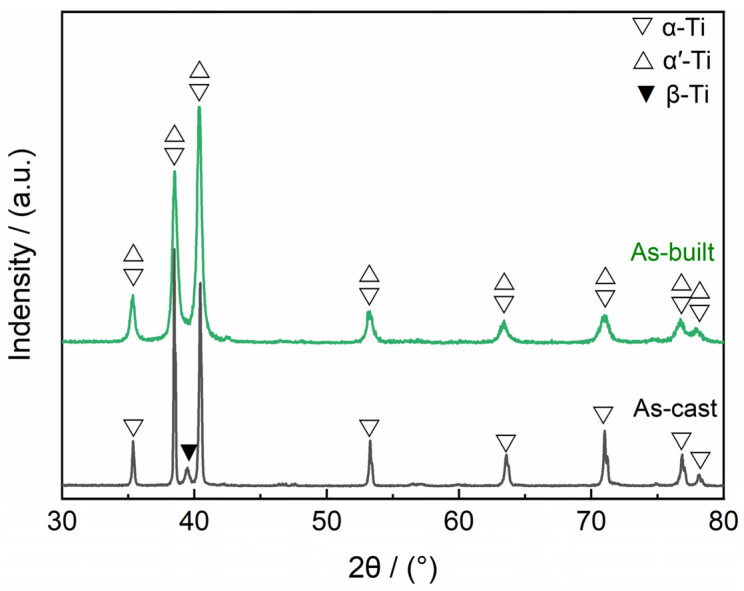
XRD patterns of the as-built and as-cast Ti6Al4V alloys.

Figure 2 shows the XRD patterns of the as-built and as-cast Ti6Al4V alloys. α, α’ and β are common phases of Ti6Al4V alloys. However, α and α’ have the same HCP lattice and similar lattice constants, so it is difficult to distinguish their characteristic peaks in XRD patterns. As shown in Figure 2, both the as-built and as-cast Ti6Al4V alloys contain α/α’ phase. Moreover, the characteristic peak of the β phase that appears near the diffraction angle of 39.5° was observed in the as-cast Ti6Al4V alloy. For the as-built Ti6Al4V alloy, no significant peaks about the β phase were detected through the XRD analysis. Similar results are also reported in other laser additive-manufactured Ti6Al4V alloys [17,18,19]. The reason is that most of the β were transformed to the α/α’ phase during the LPBF process, and the contents of the surviving β phases exceeded the detection limit of XRD.

**Figure 3:**
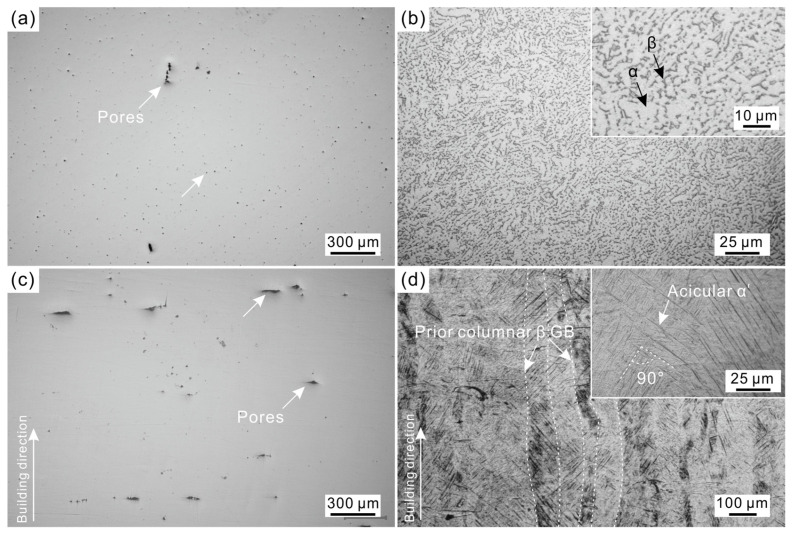
OM images of the as-cast (**a**,**b**) and as-built (**c**,**d**) Ti6Al4V alloys. (**a**,**c**) Polished surface without etching, (**b**,**d**) etched with Kroll’s reagent.

Figure 3 shows the OM images of the as-built and as-cast Ti6Al4V alloys. As shown in Figure 3a, a small number of pores can be seen on the surface of the as-cast Ti6Al4V alloy. From the etched surface morphology, a typical microstructure of the α matrix phase and the discrete β phase can be observed in the as-cast Ti6Al4V alloy (Figure 3b).

As shown in Figure 3c, the defect size of the as-built Ti6Al4V alloy is larger than that of the as-cast Ti6Al4V alloy. Metallurgical defects such as pores and lack-of-fusion voids are easily formed in additive-manufactured alloys because the additive-manufacturing process is sensitive to many factors (such as splashing, oxidation, laser energy input, melt pool stability, etc.) [20,21]. The porosity of the as-built Ti6Al4V alloy (0.53%) is higher than that of the as-cast Ti6Al4V alloy (0.35%). The microstructure of the as-built Ti6Al4V alloy exhibits significantly different morphologies than the as-cast Ti6Al4V alloy, as shown in Figure 3d. Prior columnar β grain boundaries can be seen in the as-built Ti6Al4V alloy. The prior columnar β grains are parallel to the building direction with a width size of 50~120 μm. During the LPBF process, the temperature gradient is large (10^7^ K/m) and the heat flow direction is parallel to the building direction [22]. Grains with a growth direction parallel to the heat flow direction preferentially grow, forming columnar grains, which is a typical grain growth mode in the LPBF process. Moreover, a large number of acicular phases can be observed within the prior columnar β grains. These acicular phases exhibit parallel or orthogonal relationships with each other. This is a typical acicular α’ martensitic morphology feature in the laser additive-manufactured Ti6Al4V alloys.

**Figure 4:**
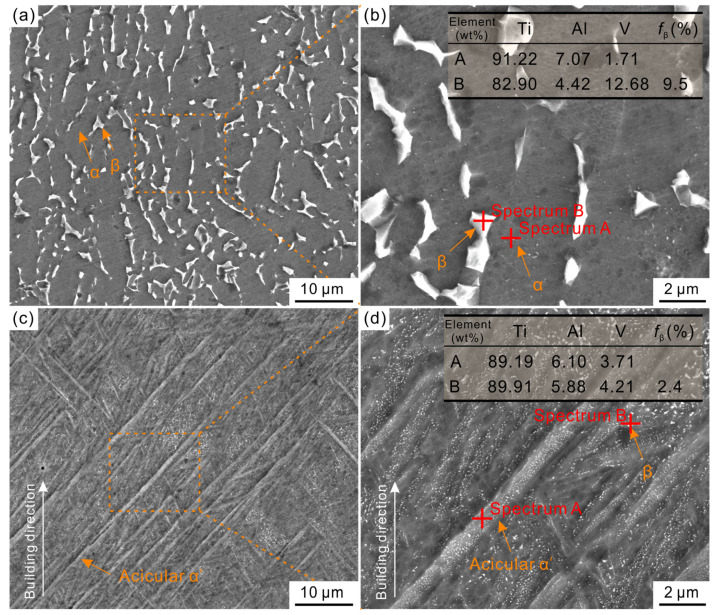
SEM micrographs of the as-cast (**a**,**b**) and as-built (**c**,**d**) Ti6Al4V alloys.

Figure 4 shows the SEM images of the as-cast and as-built Ti6Al4V alloys. As shown in Figure 4a,b, the as-cast Ti6Al4V alloy exhibits a (α + β) two-phase structure. Among them, the Al element is enriched in the α phase, while the V element is enriched in the β phase. This reveals that the alloying element is distributed towards the equilibrium condition during the traditional casting processes. As shown in Figure 4c,d, the microstructure of the as-built Ti6Al4V alloy differs from that of the traditional cast Ti6Al4V alloy in the composition and morphology of phases. The as-built Ti6Al4V alloy is mainly composed of acicular α’ martensite, in which a small amount of white granular β precipitates can be observed. The EDS results of two phases in the as-built Ti6Al4V alloy indicate that no significant difference can be detected between the α’ martensite and β phase, both of which are close to the Ti6Al4V alloy composition. This indicates that non-equilibrium solidification structures are formed due to the ultrafast cooling rate of the LPBF process. Moreover, the content of the β phase was evaluated using the Image software, the results are shown in the insets of Figure 4. The β phase in the as-cast Ti6Al4V alloy exhibits a higher volume fraction (9.5%) than in the as-built Ti6Al4V alloy (2.4%).

### 3.2. Potentiodynamic Polarization Test Results of As-Cast and As-Built Ti6Al4V Alloys

**Figure 5:**
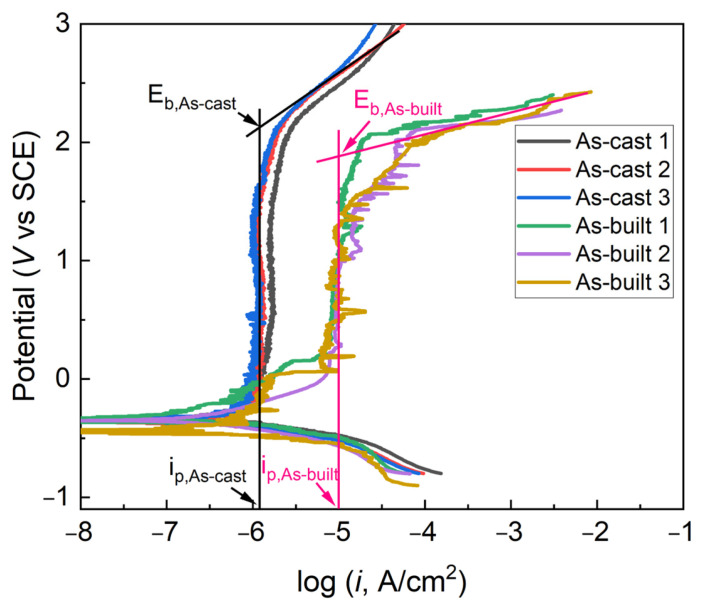
Potentiodynamic curves of Ti6Al4V alloys in 1.0 mol/L HCl solution.

As reported by most researchers [14,15], Ti6Al4V alloy exhibits excellent corrosion resistance in neutral corrosive media. So, an acidic electrolyte was chosen as the corrosion media to distinguish the differences in corrosion resistance of Ti6Al4V alloys fabricated by LPBF and casting. Figure 5 shows the potentiodynamic polarization curves of Ti6Al4V alloys in 1.0 mol/L HCl solution. Corrosion potential (*E*_corr_), corrosion current density (*i*_corr_), the pitting potential (*E*_b_) and passivation current density (*i*_p_) were fitted and are shown in Table 1. Wherein, *E*_b_ corresponds to the position where the current rapidly increases on the anodic polarization curve, and pitting corrosion occurs at this point.

As shown in Figure 5, both as-cast and as-built Ti6Al4V alloys exhibit obvious passivation characteristics, indicating that the stable passive film can be formed on their surface. However, there is a significant difference in the passivation current density and pitting potential between the two. As shown in Table 1, the *i*_p_ value of the as-built Ti6Al4V alloy is ten times those of the as-cast Ti6Al4V alloys, indicating that the as-built Ti6Al4V alloy is more difficult to be passivated in a 1.0 mol/L HCl solution. Figure 5 also shows that the as-built Ti6Al4V alloy exhibits significant current fluctuations in the passivation range, indicating its passivation film is unstable. Furthermore, the *E*_b_ value of the as-cast Ti6Al4V alloy is also greater than that of the as-built Ti6Al4V alloy, showing that the as-built Ti6Al4V alloy exhibits worse pitting corrosion resistance than the as-cast Ti6Al4V in a 1.0 mol/L HCl solution.

### 3.3. Electrochemical Impedance Spectroscopy of As-Cast and As-Built Ti6Al4V Alloys

**Figure 6:**
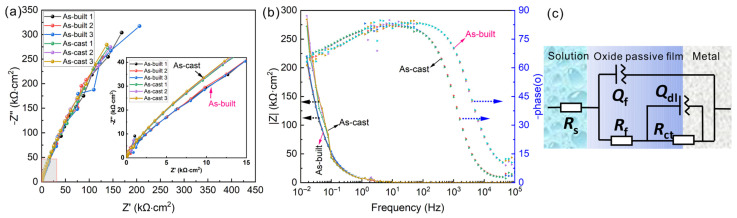
Electrochemical impedance spectroscopy for as-cast and as-built Ti6Al4V alloys in 1 mol/L HCl solution. (**a**) Nyquist plot, (**b**) bode plots and (**c**) equivalent circuit used for impedance spectra analysis. The inset figures in (**a**) are a close-up view of Nyquist plots.

To investigate the influence of semiconductor properties of passivation films on the chemical stability of Ti6Al4V alloys, the difference in corrosion resistance between the as-built Ti6Al4V alloy and as-cast Ti6Al4V alloy in a 1.0 mol/L HCl solution was evaluated using EIS. Figure 6 shows the EIS results in the form of Nyquist and Bode plots. All samples showed similar impedance characteristics, indicating the similar corrosion processes. The capacitive arc for the as-cast Ti6Al4V alloy is larger than that of the as-built Ti6Al4V alloy, suggesting that its passive film provides a higher resistance for electrochemical corrosion.

To quantitatively estimate the corrosion resistance of two samples in solution, the EIS spectra were simulated by the model R(Q(R(QR))) in Figure 6c, and the fitting results are shown in Table 2. *R*_f_ and *Q*_f_ correspond to the resistance and the capacitance of the passivation film, respectively. *R*_ct_ and *Q*_dl_ indicate the charge transfer resistance and capacitance of the double layer, respectively. Here, *Q*_f_ and *Q*_ct_ are both constant phase elements (CPE), which were used instead of the pure capacitance response. In general, *R*_ct_ can reflect the corrosion rate to a certain extent, *R*_f_ and *n*_f_ can also display the density of the passive film, the larger the *R*_f_ and *R*_ct_ values, the thinner the passive films [23,24].

As shown in Table 2, the value of *R*_ct_ for the as-cast Ti6Al4V alloy is larger than that of the as-built Ti6Al4V alloy, representing a greater electrochemical resistance and a better corrosion resistance than the as-cast Ti6Al4V alloy. In addition, the values of *R*_f_ for the as-cast Ti6Al4V alloy is 36.72 ± 2.90 kΩ·cm^2^, which is also higher than that of the as-built Ti6Al4V alloy, indicating that the density and stability of the passive film of the as-cast Ti6Al4V alloy are higher. The thickness of the passive film can be calculated for both samples by using Equation (1). Where S is the exposed area of the sample, d is the film thickness, ${ɛ}_{o}$ is the vacuum dielectric constant and its value is 8.85 × 10^−14^ F·cm^−1^, ${ɛ}_{r}$ is the relative dielectric constant of TiO_2_ passive film with a value of 48 [24] and *C* is the capacitance of passive film. Since *Q*_f_ is a non-ideal capacitance, directly inputting *Q*_f_ into *C* for calculation often results in deviation from reality. Therefore, it is necessary to use the Hus and Mansdfeld formula (Equation (2)) to calculate the effective capacitance *C*_eff_ [25,26].

(1) $$d={ɛ}_{o}{ɛ}_{r}S/C$$

(2) Ceff=Qf1nRf1−nn

The calculation results indicate that the thickness of the passive film for the as-cast Ti6Al4V alloy is 2.39 nm, but the as-built sample has a thinner passive film with a thickness of 2.02 nm. This is mainly related to the differences in organizational structure between the two. Figure 3 and Figure 4 show that the as-cast Ti6Al4V alloy exhibits a relatively uniform two-phase structure of α + β, but the as-built Ti6Al4V alloy is mainly composed of acicular α’ martensite. Research suggests that the α’ is a metastable phase and has higher electrochemical activity and a greater tendency to dissolve [13,27], which resulted in a thinner and unstable passive film for the as-built Ti6Al4V alloy.

**Figure 7:**
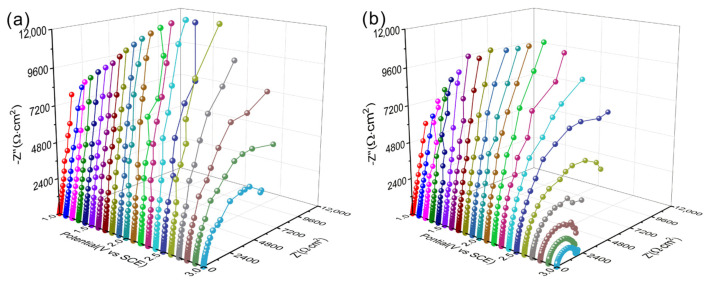
Dynamic potential electrochemical impedance spectroscopy of Ti6Al4V alloys in 1.0 mol/L HCl solution: (**a**) As-cast and (**b**) as-built.

Additionally, Figure 7 displays the dynamic potential electrochemical impedance spectroscopy (DEIS) of the as-cast and as-built Ti6Al4V alloy. The diameter of the semicircle in the impedance spectrum is related to the charge transfer resistance. As shown in Figure 7a, the diameter of the semicircle of the as-cast Ti6Al4V alloy increases with an applied potential increase from 1.0 V (SCE) to 2.1 V (SCE) and decreases when the potential is larger than 2.1 V (SCE). But for the as-built Ti6Al4V alloy, the value of the diameter firstly increases and becomes unstable when the potential is larger than 1.8 V (SCE), as seen in Figure 7b. This indicates that the stable region of passive film formed on the as-cast Ti6Al4V alloy is larger than that of the as-built Ti6Al4V alloy in a 1.0 mol/L HCl solution, which is consistent with the potentiodynamic polarization results.

**Figure 8:**
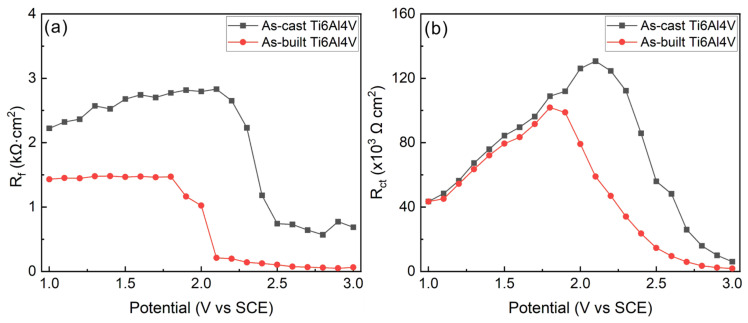
Fitting result of DEIS measurements in 1.0 mol/L HCl solution: (**a**) the passive film resistance (*R*_f_) and (**b**) charge transfer resistance (*R*_ct_).

The DEIS is also fitted using the equivalent circuit in Figure 6c since the curve pattern is similar to that at an open-circuit potential, and the fitting results of *R*_f_ and *R*_ct_ are shown in Figure 8. As shown in Figure 8, the *R*_f_ value of the as-cast Ti6A l4V alloy decreases after reaching 2.1 V (SCE), indicating a deterioration in the protective performance of the passive film and an acceleration of corrosion for the sample; the *R*_ct_ value also exhibits a similar pattern of variation. The variation pattern of *R*_f_ and *R*_ct_ values of the as-built Ti6Al4V alloy is similar, but its turning point is at 1.8 V (SCE), and both the values of *R*_f_ and *R*_ct_ for the as-built Ti6Al4V alloy are smaller than those of the as-cast Ti6Al4V alloy under different polarization potentials, indicating that the corrosion resistance of the as-built Ti6Al4V alloy in an acidic solution is worse than that of the as-cast Ti6Al4V alloy. The main reason is that the passive film on the surface of the as-built Ti6Al4V alloy is thinner (2.09 nm).

**Figure 9:**
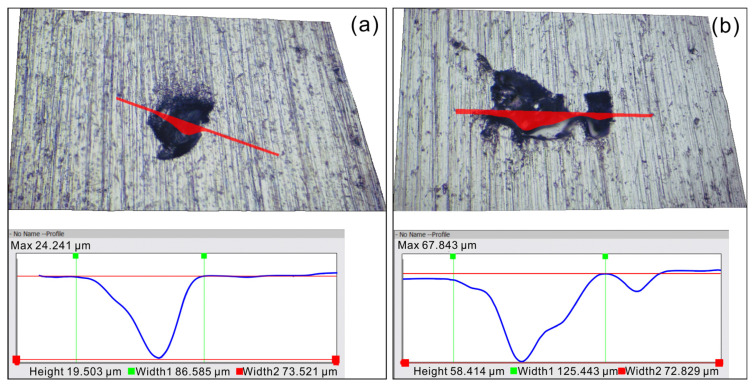
Typical morphologies and profiles of representative pits for as-cast (**a**) and as-built (**b**) Ti6Al4V alloys after DEIS testing in 1.0 mol/L HCl solution.

Meanwhile, Figure 9 shows the typical three-dimensional morphologies and profiles of corrosion pits for the as-cast and as-built Ti6Al4V alloys after conducting the DEIS tests. Overall, the size of corrosion pits formed in the as-cast Ti6Al4V alloys is small, with an average width and depth of approximately 13.0 μm and 19.5 μm, respectively. Compared with the as-cast Ti6Al4V alloy, the size of pits on the as-built Ti6Al4V alloy is generally larger. The average width and depth of pitting holes are 53.1 μm and 58.4 μm, respectively. This result indicates that the as-built Ti6Al4V alloy has a higher sensitivity and development rate of pitting corrosion than the as-cast Ti6Al4V alloy, which also effectively confirms the results of dynamic potential polarization and EIS testing.

### 3.4. Discussion on Corrosion Behavior for As-Cast and As-Built Ti6Al4V Alloys

The electrochemical test results indicate that the corrosion resistance (especially pitting corrosion resistance) of the as-built Ti6Al4V alloy is inferior to that of traditionally cast Ti6Al4V alloy in an acidic solution, the wider and deeper pits are present on its surface after the DEIS test. In general, the corrosion behavior of the alloys is closely related to the microstructure, which is influenced by the processing methods. The differences in electrochemical corrosion behavior of the as-built and as-cast Ti6Al4V alloys are closely related to the differences in defects and phase composition and morphology obtained from the two manufacturing techniques. These factors regulate the corrosion resistance of Ti6Al4V alloys by affecting the density and the thickness of the passive film on the alloy surface.

For the as-cast Ti6Al4V alloy, the slow cooling rate (10^−1^~10^0^ K/s) during the casting process results in a stable (α + β) two-phase structure [28], seen in Figure 2, Figure 3b and Figure 4a,b. However, the molten pool is the basic metallurgical unit in the LPBF process, it has the characteristics of a small size and fast movement rate, resulting in an ultrafast cooling rate (10^6^ K/s) [29]. The equilibrium diffusion of the elements is inhibited, and non-diffusive martensitic transformation occurs due to the ultrafast cooling rate of the LPBF process, the resulting non-equilibrium solidification structure of as-built Ti6Al4V alloy is occupied by acicular α’ martensite with only a small amount of β, seen in Figure 3d and Figure 4c,d. Compared to the α phase, the β phase has stronger corrosion resistance due to the passive film on its surface being more stable [13,27,30]. The content of β in the as-built Ti6Al4V alloy is only one-fourth of that in the as-cast Ti6Al4V alloy. This is one of the reasons that results in the thinner passive film (2.02 nm) of the as-built Ti6Al4V alloy.

Moreover, the matrix phase of the as-built Ti6Al4V alloy is acicular α’ martensite rather than the α phase in that as-cast Ti6Al4V alloy. Compared with the α phase, the acicular α’ martensite phase is metastable and possesses the “higher energy state” with regard to corrosion, which allows the easy dissolution of the α’ phase and results in an unstable passive film for the as-built Ti6Al4V alloy [13,27]. Moreover, the numerous defects are another important reason for the poor corrosion resistance of the as-built Ti6Al4V alloy. Due to the stress concentration at defects and the easier dissolution of the passive film at the defect site, it is highly susceptible to pitting corrosion.

The above reasons collectively lead to a thinner passive film and larger passivation current fluctuations for LPBF-processed Ti6Al4V alloy, resulting in poorer corrosion resistance. As shown in Figure 5, Figure 6, Figure 7 and Figure 8, the thickness of passive film for LPBF-processed Ti6Al4V alloy is only 84.5% of that of the as-cast Ti6Al4V alloy (2.39 nm), the passivation current density (*i*_p_) is about ten times of that of the as-cast sample and the value of *R*_f_ and *R*_ct_ are also smaller than that of the as-cast Ti6Al4V alloy.

It is worth noting that the results of this paper confirm that the corrosion resistance of LPBF-processed Ti6Al4V alloys is not as good as that of cast alloys in an HCl solution, which is consistent with previously published research findings [14,15,16]. However, this does not negate the strong potential of additive-manufactured Ti6Al4V alloy parts with complex geometric shapes. This study provides direct evidence of the relationship between the passivation performance, corrosion resistance, density and phase composition of LPBF-processed Ti6Al4V alloy. In subsequent research, the scholars can reduce the porosity, decrease the content of acicular α’ martensite phase and increase the content of β phase by improving the LPBF parameters or adding heat treatment, thereby providing a thicker surface passive film and better corrosion resistance for LPBF-processed Ti6Al4V alloy.

## 4. Conclusions

The differences in microstructure and electrochemical corrosion behavior between additive manufacturing and cast Ti6Al4V alloys were systematically researched using XRD, SEM, potentiodynamic polarization test and EIS. The following conclusions can be drawn:

(1) The LPBF-processed Ti6Al4V alloy dominates by acicular α′ martensite along with only a small amount of β-Ti phase, while the cast Ti6Al4V alloy exhibits an α + β dual-phase structure. Furthermore, LPBF-processed Ti6Al4V alloy possesses a higher porosity than cast Ti6Al4V alloy.
(2) Compared to the as-cast Ti6Al4V alloy, LPBF-processed Ti6Al4V alloy has worse corrosion resistance, especially the pitting corrosion resistance. The passive film of LPBF-processed Ti6Al4V alloy is thinner (2.02 nm) and unstable, the passivation current density is about ten times that of the as-cast sample and the value of *R*_f_ and *R*_ct_ are also smaller than that of the as-cast Ti6Al4V alloy.
(3) The poor corrosion resistance of LPBF-processed Ti6Al4V alloy should be attributed to a large number of pores, less β and a large number of acicular α′ martensite. The presence of pores disrupts the integrity of the passive film, while the presence of the acicular α′ martensite phase accelerates the dissolution of the passive film.

## Figures and Tables

**Table 1:** Corrosion parameters of the potentiodynamic polarization for Ti6Al4V alloys in 1.0 mol/L HCl solution.

Sample	*E*_corr_/V	*i*_corr_/μA∙cm^−2^	*E*_b_/V	*i*_p_/μA∙cm^−2^
As-cast	−0.341 ± 0.018	0.92 ± 0.06	2.054 ± 0.089	1.28 ± 0.34
As-built	−0.389 ± 0.046	1.11 ± 0.24	1.979 ± 0.094	11.21 ± 1.15

**Table 2:** EIS fitting parameter values of Ti6Al4V alloys in a 1.0 mol/L HCl solution.

Sample	*R*_f_ (kΩ·cm^2^)	*Q*_f_ × 10^−5^ (Ω^−1^·S^n^·cm^−2^)	*n*_f_	*R*_ct_ (MΩ·cm^2^)	*Q*_dl_ × 10^−5^ (Ω^−1^·S^n^·cm^−2^)	*n*_dl_
As-cast	36.72 ± 2.90	1.792 ± 0.089	0.980 ±0.004	1.679 ± 0.200	1.275 ± 0.009	0.991 ± 0.004
As-built	33.17 ± 1.33	2.128 ± 0.035	0.951 ± 0.003	1.469 ± 0.020	1.386 ± 0.625	0.661 ± 0.033

## Data Availability

Data are contained within this article.
